# Clinical Analysis and Literature Review of 26 Patients with Hematological Malignancies During Pregnancy

**DOI:** 10.1089/whr.2024.0164

**Published:** 2025-02-03

**Authors:** Jie Ma, Huimin Zhu, Ling Li, Hui Ma, Bo Zheng

**Affiliations:** ^1^ Department of Hematology, General Hospital of Ningxia Medical University, Yinchuan City, China.; ^2^ Ningxia rehabilitation medical center, People’s Hospital of Ningxia Hui Autonomous Region, Yinchuan City, China.

**Keywords:** hematological malignancies, pregnancy, pregnancy outcome, chemotherapy

## Abstract

**Background:**

The hematological malignancies during pregnancy are rare and lethal.

**Objective:**

The aim of this study is to demonstrate the incidence, clinical characteristics, treatment, and outcome of hematological malignancies diagnosed during pregnancy.

**Methods:**

This is a retrospective study of women who were diagnosed with hematological malignancies during pregnancy at an academic hospital between 2001 and 2021.

**Results:**

The incidence rate of 26 pregnant women with hematological malignancies in the pregnant patients admitted to the hospital was about 0.07%. Except for one patient who was diagnosed before pregnancy, the incidence rates of the first, second, and third trimesters of pregnancy were 12% (3/25), 48% (12/25), and 40% (10/25), respectively. Eleven out 26 patients (42.3%) finally gave birth to fetuses. In a long-term follow-up, the survival time of patients with leukemia in pregnancy was shorter than lymphoma (*p* < 0.05).

**Conclusion:**

This report provides a comprehensive description of maternal and infant outcomes and insights into the management of hematological malignancies during pregnancy.

## Introduction

Hematological malignancies during pregnancy refer to hematological malignancies that occurred or discovered during pregnancy. The incidence of malignancy during pregnancy is approximately 0.02%, although the incidence is relatively low, it is still one of the common causes of death in pregnant women.^1^ In recent years, with the opening of the second child and the postponement of female childbearing age in China, the incidence of hematological malignancies during pregnancy has gradually shown a rising trend. The most common hematological malignancies associated with pregnancy are lymphomas (1:1000–6000), acute leukemia (AL) (1:75000–100000), and chronic myeloid leukemia (CML) (1:100000).^2^ However, as multiple myeloma, chronic lymphocytic leukemia, myelodysplastic syndrome, and myeloproliferative neoplasms are more common in older people, so they are rarely diagnosed during pregnancy.^3^

The physiological status of pregnant patients with hematological malignancies usually changes during pregnancy.^4^ The early onset of hematological tumors during pregnancy is similar to common pregnancy reactions and blood changes under certain circumstances. So, it is easy to be confused and cause misdiagnosis, leading to delays in treatment. Though the incidence of hematological malignancies in pregnancy is lower than other pregnancy-related complications, it has a poor prognosis.^5,6^ Additionally, patients with hematological tumors in pregnancy should consider the impact on the fetus in examination and treatment. Under the premise of protecting the mother’s health and prolonging the survival time, ensuring a favorable prognosis for the fetus as much as possible has become a clinical conundrum, which needs the attention of hematologists and obstetricians.^5^ Our research retrospectively collects the clinical data of 26 patients with hematological malignancies diagnosed during pregnancy admitted to the General Hospital of Ningxia Medical University from January 2001 to February 2021. The aim of this study was to explore the clinical characteristics, diagnosis, treatment, and prognosis of pregnancy associated with hematological malignancies in order to make a useful suggestion in the management of hematological malignancies during pregnancy.

## Patients and Methods

After approval by the Research Ethics Committee, the institutional hematological malignancies database was searched for all cases of hematological malignancies and pregnancy seen from January 2001 to February 2021. A total of 26 cases of patients with hematological malignancies in the 36123 pregnant patients admitted to the hospital during the same period were identified according to World Health Organization 2008 diagnostic criteria and FAB classification. Twenty-six cases of patients with hematological malignancies in pregnancy: 15 with AL including acute myeloid leukemia (AML, *n* = 12) and acute lymphoblastic leukemia (ALL, *n* = 3), 10 with lymphoma (Hodgkin lymphoma [HL], *n* = 2) and non-HL (NHL, *n* = 8), and 1 with CML. Except for one patient with APL who was diagnosed before pregnancy, there were 3 cases in the first trimester (1–3 months), 6 cases in the second trimester (4–7 months), and 16 cases in the third trimester (8–10 months). At the same time, 100 normal pregnant patients who visited the obstetrics department of our hospital during the same period were randomly selected as the control group and compared with the hematological malignancy group.

Patient information, including age and weeks of gestational age at initial diagnosis, neoplasms types, first symptoms, initial laboratory examination (blood routine, coagulation function, immunotype, karyotype analysis, fusion-gene), treatment, pregnancy outcome (uneventful pregnancy, spontaneous or induced abortions, stillbirths, and death during childbirth), presence or absence of other pregnancy complications, and fetal status (Apgar score, presence or absence of adverse neonatal complications) were reviewed.

All 26 patients received outpatients or telephone follow-up. Patients with leukemia in pregnancy were followed up for 1–103 months, and patients with lymphoma in pregnancy were followed up for 1–87 months. The deadline for follow-up was February 28, 2021. The endpoints were death or loss of follow-up.

### Statistics

Case data and clinical data of patients were collected for statistical analysis. All data were analyzed using SPSS 26.0 software. The measurement data were expressed as mean ± standard deviation, enumeration data were expressed as a percentage (%). Categorical variables were compared using the chi-square test or Fisher’s exact test, as appropriate. The Kaplan–Meier method was used to draw the survival curve, and the log-rank test was used for survival analysis. A *p* value of <0.05 was considered statistically significant.

## Results

We identified 26 cases of hematological malignancies during pregnancy among the cohort of patients treated between January 2001 and February 2021. At diagnosis, the median age was 26 years (range 18–39 years). A summary of the patient and disease characteristics, gestational age, delivery method, therapeutic regimen, the outcomes of mother and infants are presented in Table 1.

**Table 1.** General Conditions, Characteristics, Treatment, Maternal, and Fatal Outcome in 26 Pregnant Women with Hematological Malignancies

Pt	Age	Hematological tumor type	Gestational week at diagnosis	Fusion gene	Karyotype	Pregnancy status at start of induction therapy	Anticancer drugs administered during pregnancy	Pregnancy outcome	Fetal outcome	Maternal outcome
1	29	AML-M1	37w1d	Normal	Unknown	Continue	▽IA, TA, NA	Elective c/s	Alive	Alive
2	20	AML-M2	32w2d	Unknown	Unknown	Continue	NA	Emergency c/s	Loss of follow up	Loss of follow up
3	31	APL	27w0d	PML/RARa	t(15;17)	Continue	△Arsenous acid; ▽TA	Fetal death in uterus	NA	Alive
4	33	APL	30w0d	PML/RARa	t(15;17)	Continue	▽ATRA+Arsenous acid;	Emergency c/s	Alive	Alive
5	25	APL	21w0d	NA	NA	Aborted	▽Arsenous acid; HA	TA	NA	Loss of follow up
6	30	APL	27w3d	PML/RARa	t(15;17)	Continue	△ATRA+Arsenous acid; ▽Arsenous acid	Elective c/s	Alive	Alive
7	39	APL	Unknown	PML/RARa	t(15;17)	Continue	▽Arsenous acid; TA	TA	NA	Alive
8	26	AML-M4	34w0d	NA	NA	Continue	NA	Emergency vaginal delivery	Loss of follow up	Loss of follow up
9	19	AML-M4E0	21w0d	CBFb/MYH11	Unknown	Continue	△Hydroxyurea	Death during childbirth	NA	Dead
10	28	AML-M5	22w5d	CBFb/MYH11	Unknown	Aborted	▽IA	TA	NA	Alive
11	31	AML-M5	8w0d	HOX11	Unknown	Continue	△HAA;▽TA	SA	NA	Dead
12	21	AML-M5	33w0d	NA	NA	Continue	△HA	Vaginal delivery	Alive	Dead
13	23	ALL	17w0d	NA	NA	Continue	NA	Death during childbirth	NA	Dead
14	21	ALL	26w0d	NA	NA	Aborted	NA	TA	NA	Dead
15	26	ALL	37w1d	normal	NA	Continue	▽VDLP	Elective c/s	Alive	Dead
16	26	CML	26w0d	NA	t (9; 22) (q34; q11)	Aborted	▽Hydroxyurea; Imatinib	TA	NA	Dead
17	21	NHL stage IVB	38w1d	NA	NA	Continue	▽DVP; radiotherapy	Elective c/s	Alive	Alive
18	31	NHL stage IVA	24w0d	NA	NA	Aborted	▽R-CHOP	TA	NA	Alive
19	37	NHL stage IIE	6w0d	NA	NA	Aborted	▽radiotherapy	TA	NA	Alive
20	34	NHL stage IIIA	7w0d	NA	NA	Aborted	▽CHOP	TA	NA	Loss of follow up
21	20	NHL	26w5d	NA	NA	Aborted	NA	TA	NA	Loss of follow up
22	26	NHL stage IA	24w5d	NA	NA	Aborted	▽R-CHOP; auto-HSCT	TA	NA	Alive
23	18	NHL stage IIA	33w4d	NA	NA	Continue	▽CHOP	Emergency c/s	Alive	Alive
24	38	NHL stage IIA	30w0d	NA	NA	Continue	▽R-CHEP；radiotherapy	Elective c/s	Alive	Alive
25	22	HL stage IIA	28w0d	NA	NA	Continue	▽CHOP; R-CHOP; radiotherapy	Vaginal delivery	Alive	Alive
26	25	HL stage IIAE	27w5d	NA	NA	Aborted	▽ABVD；radiotherapy	TA	NA	Alive

ALL, acute lymphoblastic leukemia; AML, acute myeloid leukemia; APL, acute promyelocytic leukemia; ATRA, all-trans retinoic acid; CML, chronic myeloid leukemia; C/S, C-section; Cy, cyclophosphamide; D, day; HAA, homoharringtonine, daunorubicin, and cytarabine; HL, Hodgkin lymphoma; NA, not applicable; NHL, non-Hodgkin lymphoma; SA, spontaneous abortion; TA, therapeutic abortion; VDLP, vincristine, daunorubicin, prednisone, and l-asparaginase; W, week.

### Clinical characteristics of hematological malignancies in pregnancy

Among 15 patients with AL in pregnancy, the clinical outcome of the initial diagnosis was anemia (13/15), bleeding from skin or mucous membrane (9/15), infection (9/15), fatigue (4/15), and chest discomfort and short of breath (2/15). In patients with APL, the bleeding from skin or mucous membrane was the main manifestation, and most patients with AML or ALL had different degrees of anemia when they were first diagnosed. A case of pregnancy with CML was presented with cervical lymphadenopathy, dizziness, and fatigue on the first visit to the outpatient clinic. Moreover, she was accompanied by mild anemia (hemoglobin: 9.60 g/dL), and an abnormal increase of white cell counts and platelets (white cell counts: 127 × 10^9^/L, platelets: 1329 × 10^9^/L). Clinical presentation of 10 pregnant patients with lymphoma varied widely according to the site of involvement. The signs and symptoms, including four cases of lymphadenopathy (two cases of cervical lymph nodes, one case of axillary lymph node, and one case of inguinal lymph node), three cases of pharyngeal masses, nasal cavity mass mediastinal mass and breast mass in one case each, other accompanying symptoms include anemia (5/10), pain (4/10), chest tightness and shortness of breath (5/10), respiratory distress and hypoxia (3/10), skin ecchymosis (1/10), and fever (1/10).

### Pregnancy management and pregnancy outcome/maternal outcomes

The median gestational age at hematological malignancy diagnosis was 26 weeks (range 7–38 weeks). Treatment was grouped as follows: received chemotherapy during pregnancy (19.2%, *n* = 5), received treatment after pregnancy termination (61.5%, *n* = 16), and gave up treatment (19.2%, *n* = 5). A total of 87.5% of the patients with AL who received treatment (*n* = 8) achieved complete remission (CR).

Eleven patients had a therapeutic induction or abortion, and 15 patients continued a regular pregnancy, of which 7 delivered at term (46.7%), 4 delivered prematurely (26.7%), 2 died during delivery with mother (13.3%), 1 spontaneous abortion (6.7%), and 1 stillbirth (6.7%). No congenital malformations were observed.

### Children outcomes

There were 11 live births, 12 abortions, and 3 intrauterine fetal death. Of the 11 live births, five were delivered by elective cesarean section, three were delivered by emergency cesarean section, two were born by vaginal delivery at term, and one was born prematurely by vaginal delivery. Five infants needed to be cared for in the neonatal intensive care unit, three due to low birth weight, and the others due to respiratory distress syndrome.

### Management of pregnancy during induction therapy

In total, 9 of the 15 patients with AL received at least one course of induction therapy. There were five patients with APL who received induction therapy with all-trans retinoic acid (ATRA), only one received chemotherapy after terminated pregnancy, and the other four patients received chemotherapy without termination (third trimester). One AML-M_1_ and one AML-M_5_ received IA (idarubicin and cytarabine), while one AML-M_5_ received HAA (homoharringtonine, daunorubicin, and cytarabine). One ALL was treated with VDLP (vincristine, daunorubicin, prednisone, and L-asparaginase). One CML received hydroxyurea before delivery and imatinib after delivery. Nine out of 10 lymphoma patients received chemotherapy or radiotherapy. Among them, two cases received chemotherapy with RCHOP (rituximab, daunorubicin, vincristine, cyclophosphamide, and prednisone) without termination, three cases received chemotherapy RCHOP while two cases received CHOP after delivery, one case received BFM90 (daunorubicin, vincristine, prednisone, and intrathecal injection of methotrexate and dexamethasone) with terminated pregnancy, and one case received radiotherapy after delivery.

### Maternal and infant follow-up outcomes

**FIG. 1.**
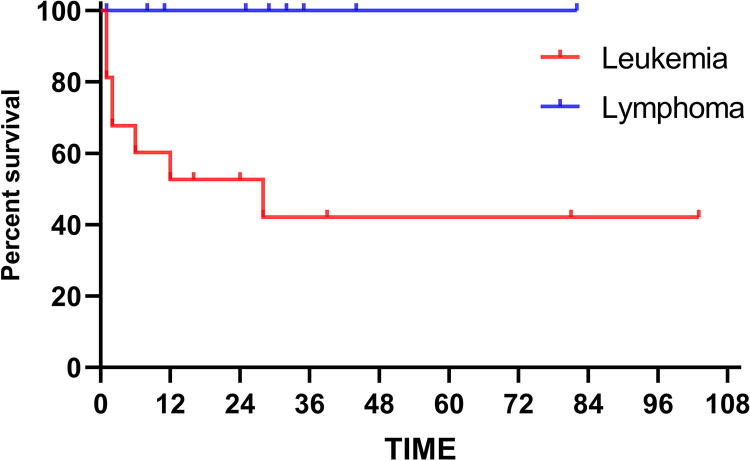
The survival rates of patients with leukemia and lymphoma in pregnancy.

Twenty-six patients were followed up for 1–103 months, and 5 patients were lost of follow-up, 8 patients died (8/21 38.1%), and 13 patients survived (13/21 61.9%). The median survival time was (72.5 ± 9.7) months, the 1-year cumulative survival rate was 74.3%, and the 3-year cumulative rate was 67.5%. Sixteen cases of pregnancy complicated with leukemia, 3 were loss of follow up, 10 cases of lymphoma with two cases lost of follow up. Comparing the survival rates of patients with leukemia and lymphoma in pregnancy, there were significant statistical differences (*F* = 5.821, *p* = 0.016), as shown in Figure 1. Nine newborns were followed up for a long-term period ranging from 1 to 146 months. By the end of the follow-up, all infants were alive at the time of the survey and demonstrated no evidence of malformation or growth retardation.

## Discussion

Although neoplasms during pregnancy are not rare, it is still uncommon in patients diagnosed with hematological malignancies during pregnancy, with an incidence rate of approximately 2 in 10,000 pregnancies. It was reported that lymphoma was the most common, followed by AL.^1^ When the patient is pregnant, standard treatment options such as radiotherapy and chemotherapy drugs may increase the risk of treatment-related adverse reactions in the mother and fetus. It is a huge challenge to ensure a good prognosis for the fetus as much as possible while protecting the mother’s health and prolonging the survival time. The emergence of molecular targeted drugs brings hope to the treatment of hematological malignancies, and there is still great room for exploration in the application of pregnant patients.

The incidence of hematological malignancies diagnosed during pregnancy has been on the rise in recent years with the increasing delay in maternal age. In this retrospective study, we described 26 cases of hematological malignancies during pregnancy at a single tertiary center in China over a period of 20 years. Our data show that the incidence of hematological tumors is about 7 in 10,000 pregnancies. Leukemia in pregnancy is dominated by AL, with AML accounting for about 2/3 and ALL accounting for 1/3.^4^ Likewise, in our study, mainly leukemia, of which AML accounts for 80% (*n* = 12), is in line with the results of foreign studies.^7^ Additionally, patients with hematological malignancies during pregnancy often have no specific clinical manifestations, so they are easily confused with normal physical changes during pregnancy, which leads to misdiagnosis. Therefore, obstetricians and hematologists should be vigilant in the diagnosis and treatment of pregnant patients.

At present, no causal relationship has been found between pregnancy and hematological malignancies.^8^ Studies have shown that hematological malignancies during pregnancy could increase the incidence of adverse pregnancy outcomes such as fetal growth restriction, which may be related to the exchange of nutrients in the villus placental interstitial space caused by leukemia cells, and damage blood flow and oxygen delivery. Moreover, leukemia cells inhibit the normal hematopoietic function of bone marrow, leading to an increased risk of maternal death, premature delivery, and stillbirth.^9^

Nowadays, the primary treatment of hematological malignancies is chemotherapy, radiotherapy, or immunotherapy. The extent of chemotherapy-induced teratogenicity is associated with the degree of drug transfer from mother to fetus, chemotherapeutic agent, drug dose, exposure duration, and maternal and child drug metabolism. Targeted therapy treatment in hematological patients during pregnancy needs further exploration.^10^ Mardi et al.^11^ successfully treated an AML during pregnancy with demethylating agent azacytidine (AZA) and sorafenib, a kind of multiple kinase inhibitors, and no adverse fetal outcomes were found after early follow-up. However, one case report could not represent the safety and efficacy of AZA and sorafenib in early pregnancy, and it needs more research to support its application in early pregnancy. It had been reported that transient neonatal myelosuppression (TNM) was seen as a rare complication in patients who received chemotherapy or rituximab administration after the 34^th^ week of pregnancy near term. In another study, it is recommended to give a 3-week interval between drug administration and anticipated delivery to avoid such outcomes.^1,12^

Pregnancy in patients with CML remains a major concern worldwide.^13–15^ Pye et al.^16^ first reported the outcomes of 180 female patients exposed to imatinib during pregnancy, outcome data are available for 125. Of those with known outcomes, 28% underwent elective terminations, 3 following the identification of abnormalities. There was a total of 12 infants in whom abnormalities were identified. Although most pregnancies exposed to imatinib are likely to have a successful outcome, there remains a risk that exposure may result in serious fetal malformations. More evidence has accumulated to indicate the potential embryotoxic and teratogenic effect of TKIs including imatinib,^17,18^ nilotinib,^19^ and dasatinib^20^ during pregnancy.

There is a great variation in the prognosis and treatment in different hematological malignancies, and it makes a great difference when to start treatment.^21^ It was reported that chemotherapy in the first trimester could result in the risk of fetal malformation, abortion, and stillbirth increased by 10%–20%.^22^ With the gestational week increased, the risk of fetal malformation caused by chemotherapy decreased significantly, and it was similar to the general population with an incidence of teratogenicity about 3% in the second and third trimesters. Horowitz et al.^23^ demonstrated that receiving chemotherapy during pregnancy would reduce fetal survival rate, according to a cohort AML patients divided into two groups with or without receiving chemotherapy during pregnancy. In Nosha’s study,^7^ 15 patients during the second or third trimester of pregnancy received regular chemotherapy, of which no additional increase in fetal malformation. In our study, we found one AML-M5 patient underwent a spontaneous abortion after receiving a course of HAA during the first trimester, considering it may be related to the use of cytotoxic drugs in the early stages of pregnancy. Therefore, chemotherapy in the first trimester still needs to be cautious. APL is a special subtype with better prognosis in AL, and patients with APL during pregnancy have a high possibility to achieve CR and even can be cured.^24^ ATRA and arsenic can significantly improve the prognosis of APL, but arsenic could cause heavy toxic effects on the fetus in the first trimester, such as damage to the central nervous system and cardiovascular malformation.^8^ Some researchers suggested that they could receive ATRA monotherapy if the patients who were in the third trimester had a strong intention to receive chemotherapy.^25^ We found that two APL patients received ATRA with or without arsenic in the third trimester, of which one case had a healthy baby and the other one experienced intrauterine fetal death. It is considered to be related to the arsenic. The remaining three patients with APL chose induced labor and started treatment after termination of pregnancy. All of them, except for one who was lost to follow-up, showed a favorable prognosis. Similar cases have been reported overseas.^24^ Patients with APL during pregnancy can get a relatively good prognosis after treatment. For patients with lymphoma, they usually need to be treated with a combination of chemotherapy and radiotherapy.^1^ Fetal exposure to radiation resulted in intrauterine growth retardation, malformation, and stillbirth. Otherwise, radiotherapy can cause severe, irreparable DNA damage, leading to mental retardation, cataract, tumors, and so on. Thus, radiotherapy during pregnancy requires extra caution. Chelsea et al.^26^ performed a study with 39 patients with lymphoma during pregnancy, in which 24 of the cases received prenatal chemotherapy. Only 10.3% of patients experienced a spontaneous abortion (*n* = 4), and the others had no serious malformation. It has been proven that received treatment for patients with lymphoma during the late trimester was relatively safe. Some scholars have proposed that for some patients, with no symptoms, stage IA/B, and stage IIA except for mediastinal lymphoma, especially diagnosed during a late trimester, were advised to delay chemotherapy and radiotherapy until delivery.^27^ In our research, patients with lymphoma diagnosed during early trimester chose to terminate pregnancy and received treatment, whereas patients diagnosed during late trimester chose to receive treatment after delivery. All of them had a long-term survival.

Of the 14 patients who chose to continue their pregnancy in the present study, 35.7% (*n* = 5) had premature births, 57.1% (*n* = 8) had cesarean section, 7.1% (*n* = 1) had postpartum hemorrhage, and 28.6% (*n* = 4) had low weight infants. Compared with control group (100 normal pregnant patients), there was no significant difference in premature birth rate, cesarean section rate, postpartum hemorrhage rate, and incidence of low birth weight infants. Su Eryun^28^ and others in China collected 152 patients with AL during pregnancy reported in the Chinese literature, there were about 27% of the fetuses died, 19.7% had miscarriages, and about 90% of the 52 patients who received chemotherapy during pregnancy had premature births. It is considered that AL would lead to the incidence of adverse pregnancy outcomes. The current treatment of hematological malignancies during pregnancy remains controversial. To exactly evaluate the prognostic impact of chemotherapy during pregnancy, research described long-term follow-up for physical, psychological, and learning abilities of 84 children who were exposed to chemotherapeutic agents *in utero*. Among them, 38 were born after receiving chemotherapy in the first trimester. However, another study found that receiving chemotherapy during pregnancy would increase the incidence of adverse fetal outcomes, and about 17% of babies had malformations. Hence, they suggested that children with a history of chemotherapy or radiotherapy exposure during pregnancy had a higher risk of breast cancer and thyroid cancer.^22^ In this article, we performed long-term follow-up on newborns. Except for two who were lost to follow-up, nine children survived and, without physiological or psychological anomalies, including two cases were exposed to chemotherapy in the uterus.

AL during pregnancy was more frequently found in AML, and ALL was rare.^11^ Related literature revealed that ALL during pregnancy had a poor prognosis and recommended to terminate the pregnancy immediately after initial diagnosis and initiate therapy. Our data showed that all three cases of ALL during pregnancy had a poor prognosis. However, the results lack representation, due to the small sample size. Patients with APL during pregnancy showed a better prognosis than other types of leukemia.^8,29^ Our five patients all achieved CR and observed long-term survival. Compared to patients with NHL during pregnancy, patients with HL during pregnancy had a better prognosis. Furthermore, it had no additional risk of fetal malformation or stillbirth and had a similar survival rate compared with normal pregnancy.^30,31^ Survival analysis of 16 patients with leukemia and lymphoma in pregnancy in this article found that the survival rate of patients with leukemia was significantly lower (*p* < 0.05). The survival rate of patients with leukemia, with the exception of APL, was lower than that of lymphoma. Comparing the average survival time, cumulative survival rate, and pregnancy outcome of patients receiving chemotherapy with or without termination of pregnancy, a higher incidence of adverse pregnancy outcomes was observed in chemotherapy during pregnancy. In contrast, chemotherapy during pregnancy did not have a significant impact on the CR rate of patients and the risk of fetal teratogenesis did not increase.

## Conclusion

Although the incidence of hematological malignancies in pregnancy is relatively low, it is still one of the common causes of death in pregnant women. In recent years, as the age of pregnant women continues to delay, the incidence of hematological malignancies during pregnancy is also increasing. When the patient is pregnant, standard treatment options such as radiotherapy and chemotherapy drugs may increase the risk of treatment-related adverse reactions in the mother and fetus. It is a huge challenge to ensure a good prognosis for the fetus as much as possible while protecting the mother’s health and prolonging the survival time. However, the emergence of molecular targeted drugs and cellular immunotherapy has broad prospects for the future development of hematological malignancies. There is still much room for exploration in the treatment of hematological malignancies in pregnancy.

In this study, we analyzed the clinical data of 26 pregnant women with hematological malignancies, and at the same time, we summarized and discussed the literature review. The findings of this study may increase awareness with regard to hematological tumors during pregnancy, leading to improved clinical treatment and diagnosis.

## Abbreviations Used

AL: acute leukemia
ALL: acute lymphoblastic leukemia
AML: acute myeloid leukemia
APL: acute promyelocytic leukemia
ATRA: all-trans retinoic acid
AZA: azacytidine
CML: chronic myeloid leukemia
CR: complete remission
HL: Hodgkin lymphoma
HAA: homoharringtonine, daunorubicin, and cytarabine
NHL: non-Hodgkin lymphoma
RCHOP: rituximab, daunorubicin, vincristine, cyclophosphamide, and prednisone
VDLP: vincristine, daunorubicin, prednisone, and l-asparaginase
